# Characterization of Comb-Shaped Copolymers by Multidetection SEC, DLS and SANS

**DOI:** 10.3390/polym9020061

**Published:** 2017-02-14

**Authors:** Giulia Gelardi, Nicolas Sanson, Gergely Nagy, Robert J. Flatt

**Affiliations:** 1 Institute for Building Materials, ETH Zurich, CH-8093 Zurich, Switzerland; gelardig@ethz.ch; 2 Soft Matter Sciences and Engineering Laboratory, CNRS, ESPCI Paris, PSL Research University, 10 rue Vauquelin, 75005 Paris, France; nicolas.sanson@espci.fr; 3 Soft Matter Sciences and Engineering Laboratory, Université Pierre et Marie Curie, Sorbonne-Universités, 10 rue Vauquelin, 75005 Paris, France; 4 Laboratory for Neutron Scattering and Imaging, Paul Scherrer Institute (PSI), CH-5232 Villigen, Switzerland; gergely.nagy@psi.ch or gergely.nagy.risp@gmail.com

**Keywords:** comb-shaped copolymers, superplasticizer, solution conformation, radius of gyration, multidetection size-exclusion chromatography, dynamic light scattering, small-angle neutron scattering

## Abstract

PolyCarboxylate ether-based superplasticizers (PCEs) are a type of comb-shaped copolymers used as polymeric dispersants in cementitious materials. PCEs have a high degree of dispersity, which limits the suitability of conventional characterization techniques, such as Size Exclusion Chromatography (SEC). Properties of PCEs strongly depend on their molecular structure and a comprehensive characterization is needed to fully understand the structure–property relationships. PCEs with well-defined molecular structures were synthesized to study their solution conformation by SEC and scattering techniques. The combined use of SEC, dynamic light scattering and small-angle neutron scattering allowed us to demonstrate the validity of a scaling law describing the radius of gyration of comb-shaped copolymers as a function of their molecular structure. Moreover, we show that the use of SEC with standard calibration, although widely spread, is not adequate for PCEs.

## 1. Introduction

Comb-shaped copolymers are complex polymers, consisting of a main chain (or backbone) onto which side chains of a different chemical nature are grafted. Comb-shaped copolymers with an anionic backbone have been used as dispersants for the stabilization of various types of suspensions, such as cement and concrete [1,2], limestone [3], silica [4], gypsum [5], barium titanate [6], and magnesia [7]. Indeed, the negatively charged backbone first adsorbs onto the positively charged surfaces by electrostatic interactions, whereas the dispersion itself is due to the non-adsorbing neutral side chains inducing steric hindrance between the polymer coated surfaces [8,9].

In the field of cementitious materials, the comb-shaped copolymers used in this work are referred to as PolyCarboxylate Ether (PCE) superplasticizers. The addition of PCEs to cement and concrete enhances the workability and allows the reduction of the amount of mixing water, which increases strength and improves durability [10]. PCEs can also play an essential role in reducing the carbon footprint of concrete [11]. Consequently, they are very largely used in practice—(about two million tons per year). 

Many different types of PCEs for concrete applications are available on the market [12]. While the side chains are usually made of monofunctional polyethylene glycol, various types of monomers can be used for the synthesis of the backbone. The great flexibility in the design of PCEs led to a very active research directed towards the synthesis of polymers with different chemical characteristics and performances [13,14,15]. The types of PCE used in this work consist of a backbone of polyacrylic or polymethacrylic acid (PAA or PMA) and side chains of methoxy polyethylene glycol (MPEG), which are the most used in practice.

Properties of PCEs depend on their molecular structure. Over the last years, important progress has been made in understanding structure–property relationships, also for what concerns the effect on cement hydration or rheology [16,17]. Such progress suggests that molecular design of PCEs to design products with targeted performances is possible [18,19].

Understanding structure–property relations is however a complex objective because PCEs are highly polydisperse. Within a single polymer, fractions with different molecular characteristics (e.g., length of backbone, and grafting density) are present. The method of choice for the characterization of PCEs is Size Exclusion Chromatography (SEC). However, SEC cannot resolve peaks within the polymeric fraction, and co-elution of species can often occur [20]. The proper interpretation of SEC is therefore an important open question that is also very relevant for two-dimensional chromatography, which represents a promising perspective for characterizing comb copolymers [21].

Several studies have shown how the performance of PCEs is affected by their average molecular structure [22,23,24,25]. However, the role of dispersity, formerly known as polydispersity, remains poorly quantified and requires further investigation. In particular, the development of adequate analytical tools represents an essential step to understand which parts of the PCEs are responsible for the different properties.

Besides their dispersity, an additional challenge for characterizing PCEs is that they are charged polymers. In particular, ionic interactions between the polymer chains and the stationary phase of the SEC column can cause elution of the polymer in non SEC-mode [20]. To screen the electrostatic charges salts need to be added to the polymer solution. In this study, two types of electrolytes have been used: disodium phosphate and calcium chloride. The first has been reported as a good mobile phase for size exclusion chromatography of linear polyacrylic and polymethacrylic acids [26], whereas the second has been selected to represent typical concentrations of calcium in the aqueous phase of hydrating cementitious systems. 

In the present work, we have synthesized comb copolymers with a narrow dispersity, by grafting side chains onto previously synthesized backbones. By producing PCEs by grafting-onto method rather than copolymerization, the size of the backbone can be reliably determined first. Combining this information with independent measurement of the grafting density and the size of the side chains, we produced model copolymers for which all the molecular structure parameters were known. With such polymers at hand, it was possible to relate the behavior of PCEs in SEC to the PCE molecular structure. 

Moreover, by using additional methods as dynamic light scattering and small-angle neutron scattering, we demonstrated that SEC provides size information that is consistent with a polymer physics based scaling law for comb copolymers conformation in solution [27,28]. In particular, it is shown that the molar mass determination based on the widely-spread approach of calibration with polyethylene oxide and polyethylene glycol (PEO/PEG) standards is not reliable. As a premise to this study, we therefore present a summary of the theoretical approach leading to a scaling relation for the conformation of comb copolymers in solution.

### Conformation in Solution: A Theoretical Approach

**Figure 1:**
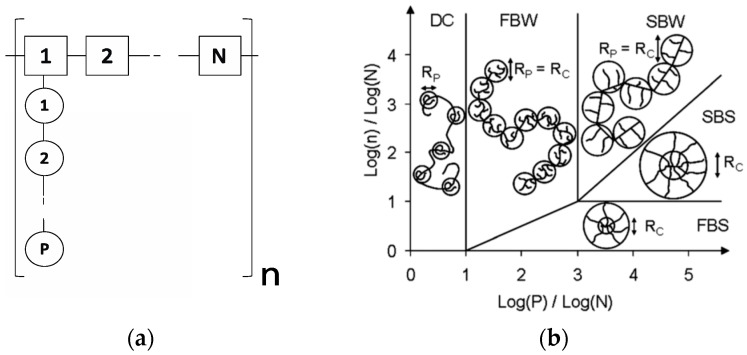
(**a**) Schematic representation of a comb polymer made of *n* repeating units, each containing *N* monomers in the backbone and one side chain of *P* monomers. (**b**) Phase diagram derived by Gay and Raphael for comb homopolymers: DC = decorated chain, FBW = flexible backbone worm, SB = stretched backbone worm, SBS = stretched backbone star, FBS = flexible backbone star [27].

An important aspect of the working mechanism of PCE is their conformation, both in solution and at interfaces. Conformation of PCEs in solution has been described by the model proposed by Gay and Raphael, initially derived for comb homopolymers in a good solvent [27]. According to this model, the polymer is made of n repeating units, each containing N monomers in the backbone and one side chain of P monomers, as represented in Figure 1a. Depending on the relative magnitude of these parameters, the polymers can assume different conformations, as shown in Figure 1b.

Among the possible conformations for comb homopolymers in solution, the Flexible Backbone Worm (FBW) conformation is the most relevant for PCEs used in practice and corresponds to a self-excluding chain of *n*/*n*_C_ blobs (where *n*_C_ is the number of side chains in a blob) and each blob has a radius *R*_C_. The overall radius of gyration, *R*_G_, follows the Flory scaling law [29].

This model has been extended to describe the radius of gyration of comb copolymers by Flatt et al. The equation can be written as:

(1) $$R_{G}={\left({\left(\frac{a_{N}}{a_{P}}\right)}^{2}\frac{\left(1-2\chi \right)}{2}\right)}^{1/5}a_{P}P^{2/5}N^{1/5}n^{3/5}$$

where *a*_N_ and *a*_P_ are the size of the backbone and side chain monomers, respectively, and χ is the Flory parameter. For the PCEs used in this work, *a*_N_ = 0.25 nm, *a*_P_ = 0.36 nm and χ = 0.37 [28].

For the calculations, the structural parameters *P*, *N* and *n* are needed. The parameter *P* is defined by the molar mass of the side chains and is usually known *a priori*. On the other hand, *N* and *n* need to be determined. The parameter *N* is related to the grafting density and can be determined by the stoichiometry of the reaction and the degree of reaction or by ^1^H-NMR. For what concerns the structural parameter *n*, it is related to the length of the backbone. For polymers produced by a grafting-onto method (polymer analogous esterification), it is possible to first determine the backbone length, *n × N*, from a SEC measurement. However, if the polymers are produced by copolymerizing backbone monomers with macromonomers bearing the side chains, this procedure is not applicable. Standard practice relies on SEC of the PCEs, using PEO/PEG as molar mass standards, which we will show below to be inadequate. Another drawback of copolymerization is that it introduces a larger dispersity in the grafting density *N* because of the different reactivity of the two monomers [30,31]. In contrast, comb copolymers prepared by grafting can be expected to have a much more homogenous distribution of the side chains along the backbone. Indeed, the grafting-onto reaction should lead to close to equilibrium conditions, so that energy minimization would homogenize the side chain distribution.

A further factor of dispersity can be the side chains. In this study we used methyl terminated polyethylene glycol (MPEG) side chains that are typically found on the market. Conveniently, commercially available MPEG is rather monodisperse (dispersity, *Đ* = *M*_w_/*M*_n_ ≤ 1.1), so that *P* is taken as a single value. 

Overall, this implies that for PCEs prepared by grafting, the main factor of dispersity arises from the backbone. This is why we focused first on producing relatively monodisperse backbones for this study, thereby obtaining a set of model comb copolymers well suited for studying possibilities and limitations of PCE characterization by SEC and the relation to the theoretical conformation model described above. Additionally, PCEs that are closer to those used in practice (e.g., broad molar mass distribution of both backbone and PCE) were also used in this work.

## 2. Materials and Methods

### 2.1. Materials 

Polyacrylic acid (*M*_w_ ~ 5000 g/mol, Polysciences, Warrington, PA, USA), methoxy polyethylene glycol (*M_n_* ~ 2000 g/mol, Sigma-Aldrich, Buchs, Switzerland), deuterium oxide (D_2_O, deuterium content 99.9%, Sigma-Aldrich), sodium phosphate dibasic (Na_2_HPO_4_, for HPLC, ≥98.5%, Sigma-Aldrich), and calcium chloride dehydrated (CaCl_2_, >97%, Sigma-Aldrich) were used as received.

### 2.2. Synthesis of PCEs

The synthesis of the comb-shaped copolymers was realized by esterification of the carboxylic groups of pre-formed backbones of polyacrylic acid or polymethacrylic acid. 

Three types of backbone were used for the synthesis of PCEs: polyacrylic acid backbones prepared by Reversible Addition Fragmentation Chain Transfer (RAFT) polymerization (Table 1, A1–A4), polymethacrylic acid backbones prepared by radical polymerization (Table 1, B1–B6) and commercial polyacrylic acid backbones (Table 1, C1–C4). These backbones differ in their chemical nature, average molar mass and degree of dispersity (Table 1). 

More details about the synthesis of the PAA backbone via RAFT polymerization can be found in the Supplementary Materials.

The esterification of PAA backbones with MPEG was performed at 165 °C under reduced pressure (80 mbar). Different grafting densities were reached using various amount of MPEG or different reaction times. After the esterification the reaction mixture was ultrafiltered by using polymeric membranes (Pellicon XL 50 Cassette, Merck Millipore, Schaffhausen, Switzerland) with a cut-off of 10 kDa. The complete removal of the side chains was confirmed by SEC.

The PCEs labeled as B1–B6 were provided by Sika Technology (Zurich, Switzerland). For these, the value of grafting density was provided by the company. The method applied involved the use of high performance liquid chromatography to determine the amount of unreacted side chains. 

For each PCE, a theoretical molar mass (*MM*^theor^) was calculated using the known molar masses of backbone and side chain and the grafting density. 

### 2.3. Proton Nuclear Magnetic Resonance (^1^H-NMR)

All the NMR measurements were performed on a 300 MHz Bruker spectrometer. ^1^H-NMR was used to confirm that esterification of the backbone has taken place and to determine the grafting density of PCEs (A1–A4, and C1–C4). More details about this can be found in the Supplementary Materials.

### 2.4. Multidetection Size Exclusion Chromatography (SEC)

Size exclusion chromatography was performed on an Agilent 1260 Infinity system (Agilent Technologies, Santa Clara, CA, USA) equipped with a refractive index detector (Agilent), an ETA-2010 Viscometer (PSS, Polymer Standards Service GmbH, Mainz, Germany) and a SLD7000 on-line multi-angle light scattering detector. A series of one PSS Suprema column (0.8 cm × 30 cm, particle size 10 μm) with a pore size of 30 Å and two PSS Suprema columns (0.8 cm × 30 cm, particle size 10 μm) with a pore size of 1000 Å was used. The mobile phase used was 0.1 M Na_2_HPO_4_ solution. This mobile phase was prepared by using ultrapure water from a Millipore Milli-Q system (TOC < 2 ppb). The flow-rate used was 1 mL/min. Data analysis was performed using WinGPC Software by PSS.

Polymer solutions (concentration range: 2–5 g/L) were prepared in the solvent used as mobile phase and left to dissolve overnight.

A series of PEO/PEG molar mass standards (232 g/mol < *M*_w_ < 1000 kg/mol, PSS) were used to generate a calibration curve for the SEC system and determine the relative molar mass of PCEs. 

The absolute molar mass of the PCEs was also determined by SEC-MALS (Multiple Angle Light Scattering). Angle normalization and determination of the inter-detector delays and detector response factors were performed by using a Pullulan sample having *M*_w_ = 112,000 g/mol and low dispersity (*Đ* = 1.13), purchased at PSS. The refractive index increment (*d*n/*d*c) of the PCEs was determined online and found to be in the range 0.135–0.145 mL/g.

The PAA backbones synthesized by RAFT polymerization were also characterized by SEC and SEC-MALS (see Supplementary Materials).

The intrinsic viscosity of the PCEs was determined on-line by SEC-Viscometry (SEC-VISC). The inverse of the intrinsic viscosity was taken as a first estimate of the overlap concentration for each PCE [20]. The calculated overlap concentrations are reported in the Supplementary Materials (Table S1).

### 2.5. Dynamic Light Scattering (DLS)

Dynamic light scattering measurements were performed on a Zetasizer Nano instrument (Malvern, Herrenberg, Germany). Polymer solutions (about 2 g/L) were prepared in Na_2_HPO_4_ 0.1 M and CaCl_2_ 20 mM and filtrated through 0.45 μm pore-sized filters. The polymer concentration chosen is between 7 and 35 times smaller than the overlap concentration estimated from the intrinsic viscosity. Moreover, additional DLS measurements were carried out at lower concentrations (about 0.5 g/L) and results are shown in the Supplementary Materials (Figure S3). The samples were stabilized at constant temperature for 1 min prior to measurement. All the measurements were carried out at a temperature of 25 °C. Examples of the correlation functions of PCEs can be found in the Supplementary Materials (Figure S4).

The values of the hydrodynamic radii were calculated from the decay times using Stokes–Einstein equation: *R*_H_ = *k*_B_*T*/6πη*D* where *k*_B_ is the Boltzmann constant, *T* is absolute temperature, η is the solvent viscosity and *D* the coefficient diffusion.

### 2.6. Small-Angle Neutron Scattering (SANS)

The SANS measurements were performed at the Paul Scherrer Institute (Villigen, Switzerland) on the SANS-II instrument [32]. The neutrons were detected with a ^3^He detector. 

The measurements were performed using three combinations of neutron wavelength, λ, and sample-to-detector distance, d, (i.e., λ = 10.6 Å, *d* = 6 m; λ = 5.3 Å, *d* = 4 m, λ = 5.3 Å, *d* = 1.2 m), giving a *q*-range of 0.02 nm^−1^ ≤ *q* ≤ 2.5 nm^−1^.

Polymer solutions (about 10 g/L) in CaCl_2_ 20 mM and Na_2_HPO_4_ 0.1 M in D_2_O were freshly prepared and placed in 1 mm thick quartz cuvettes. The measurements were carried out at room temperature (*T* = 25 °C).

Data reduction was done with Grasp software (C. Dewhurst, ILL, Grenoble, France) and fitting with SASfit [33]. Examples of the SANS curves of PCEs can be found in the Supplementary Materials (Figure S5).

## 3. Results

### 3.1. Multidetection Size Exclusion Chromatography

**Figure 2:**
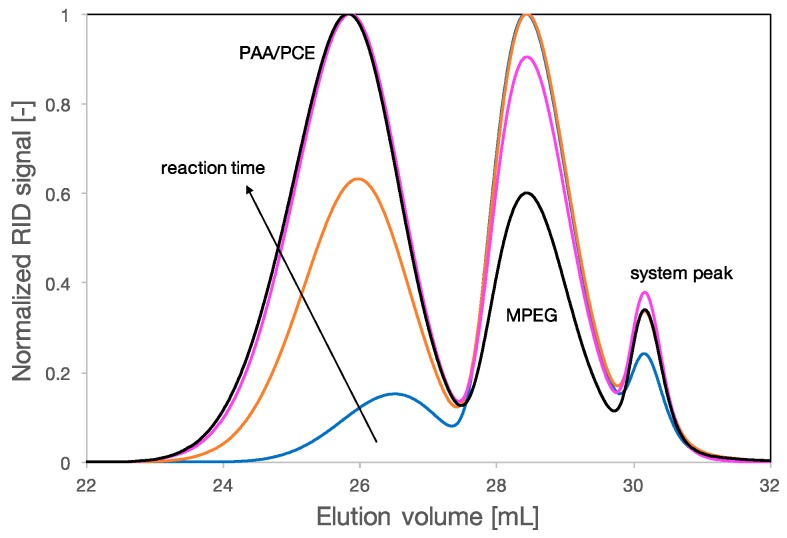
Chromatograms of reaction mixture (PAA, MPEG and PCE) at different times: 0 h (blue), 1 h (orange), 1.5 h (pink), and 2 h (black). The first peak is the PCE (only PAA at 0 h) showing a progressive increase of molar mass due to the ongoing grafting onto the backbone, the second peak is the MPEG and decreases in intensity with time since it is consumed during the reaction. The last peak at around 30 mL is the system peak.

Samples of the reaction mixtures were taken at different times and analyzed by SEC. The chromatograms relative to the grafting of MPEG onto a PAA backbone are shown in Figure 2. 

Due to the difference of elution volume, *V*_e_, between the backbone, the MPEG side chain and the final PCE, SEC allows to clearly identify the varying proportions of reagents and products during the synthesis. Before the reaction starts, the backbone is the first eluting species. As the reaction occurs, its elution volume shifts to lower values and higher intensities, revealing the progress of the grafting onto the backbone. Additionally, the intensity of the peak of the MPEG, at about 28.5 mL, decreases as the MPEG is consumed during the reaction. The fact that the esterification indeed took place was confirmed by ^1^H-NMR (Figure S1, Supplementary Materials).

**Figure 3:**
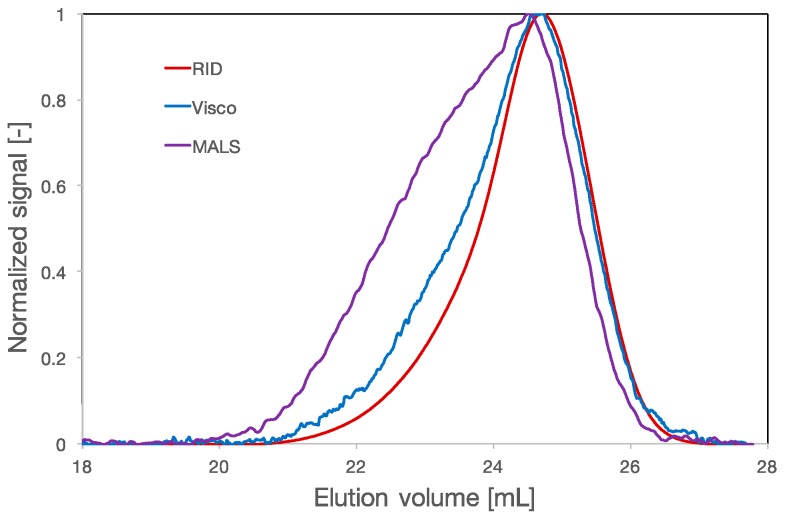
Chromatogram of the PCE labeled as A2. The red line is the signal of the refractive index detector, the blue line is the viscosity detector signal and the purple line is the signal of the multi-angle laser light scattering detector.

Multidetection SEC was used to analyse the ultrafiltered PCEs. For some of the polymers (Figure 3), shouldering of the peak in the MALS signal can be observed. However, the signal of the refractive index detector does not reveal any shoulder. This suggests that there are polymeric fractions of larger molar mass at very small concentration, for example polymers resulting from the crosslinking of PCE molecules. Crosslinking may occur during the esterification reaction when the side chains are not mono-functional but rather diols and can react with the carboxylic function of two different backbones, leading to the formation of a larger molecule.

To determine the molar mass distributions from the type of SEC measurements reported above, two different approaches were used: (i) calibration based on PEO/PEG molar mass standards; and (ii) using MALS detector to obtain the absolute molar mass.

**Figure 4:**
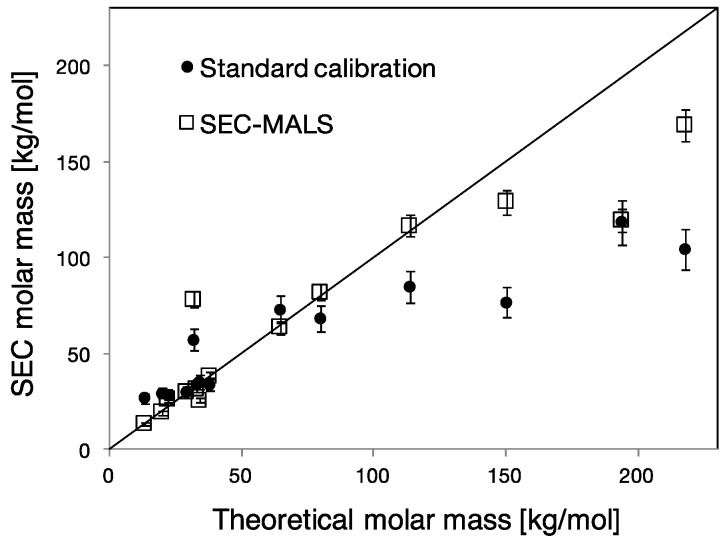
Weight-average molar mass of PCEs determined by standard calibration (filled circles) and SEC-MALS (squares) as a function of theoretical molar mass. The error bars represent the typical error of molar mass determination via standard calibration (±10%) and SEC-MALS (±5%).

The weight-average molar mass (*M*_w_) of the synthesized PCEs, as determined by both standard calibration and SEC-MALS, is reported as a function of the theoretical molar mass in Figure 4. The straight line represents a 1:1 relation between the theoretical and the experimental molar masses. This theoretical mass is determined from the backbone molar mass, the grafting density and the molar mass of the side chains. Since the structural parameters of our PCEs are either known a priori or reliably measured, we assume that this theoretical molar mass is a good estimate of the “true” molar mass. The graph in Figure 4 shows that SEC-MALS better matches the theoretical mass.

### 3.2. R_H_, R_G_ and R_η_ of Polycarboxylate Ethers

Hydrodynamic radii between 6 and 17 ± 1 nm were obtained by DLS. The error on the hydrodynamic radii is the standard deviation based on replicate measurements. The *R*_H_ values are reported in Table 2.

The radii of gyration of PCEs were determined by fitting the SANS curves using the Debye equation, which relates the scattering intensity to the radius of gyration *R*_G_ of the polymer as follows:

(2) $$I\left(q\right)=\frac{2I_{0}}{q^{4}R_{G}^{4}}\left(e^{-q^{2}R_{G}^{2}}-1+q^{2}R_{G}^{2}\right)$$

where *I*_0_ is the forward scattering intensity at zero angle. Guinier and Zimm models were also used to fit the SANS data and provided similar values as those obtained by Debye equation. The latter was preferred since it was already used in another study involving PCEs [34]. The calculated *R_G_* values are shown in Table 2. The error on the *R*_G_ values is estimated to be ±0.1 nm. With the *R_G_* values obtained by SANS, we have calculated the overlap concentration of the PCEs, defined as C* = [(3*M*)/(4π*R*_G_^3^)]. The overlap concentrations were found to be between 15 and 88 times higher than the concentration used for the SANS experiments, indicating that all scattering experiments were carried out in the dilute regime. The newly calculated overlap concentrations are reported in the Supplementary Materials (Table S1).

The viscometric radii, *R*_η_, were calculated from the intrinsic viscosities, [η], which were measured by SEC-VISC. The equation that relates the two parameters is Einstein’s expression for hard spheres:

(3) Rη=(3[η]M10πNA)13

The calculated values of *R*_η_ are reported in Table 2. The error on the viscometric radii is estimated to be ±0.1 nm and represents the standard deviation based on replicate measurements.

The values reported in Table 2 refer to experiments carried out in Na_2_HPO_4_ 0.1 M for DLS and in CaCl_2_ 20 mM for SANS. The DLS and SANS measurements carried out in different solvents lead to similar results. Since the solvent does not seem to substantially affect the investigated solution properties of PCEs, the other results are not discussed.

## 4. Discussion

### 4.1. Molar Mass Determination of PCEs

Standard calibration based on PEO/PEG linear polymers is the method largely used in practice for the determination of the molar mass of PCEs. The choice of using PEO/PEG standards is generally justified by advocating that most of the mass (about 80%–90%) of a PCE molecule is given by PEG side chains. However, it is known the standard calibration only provides relative molar mass values for analytes that are chemically different or have different structures from the standard [20]. 

Figure 4 shows that, in most cases (in particular at high molar masses), standard calibration leads to the underestimation of the molar mass of the comb copolymer. To address this issue quantitatively, we must consider that SEC is used to determine a molar mass, but that the primary information that it delivers is related to the size of polymers in solution. In other words, rather than using the molar mass of the standards to calibrate the system, it would be better to use their hydrodynamic radius, *R*_H_. For our PEO/PEG standards, both properties are related through the power law relationship for linear chains reported below:

(4) $$R_{H}=a\;{M_{w}}^{b}$$

where *a* has magnitude 0.0145 and units of nm (mol/g)*^b^* and *b* is 0.571, according to Devan and Selser [35].

**Figure 5:**
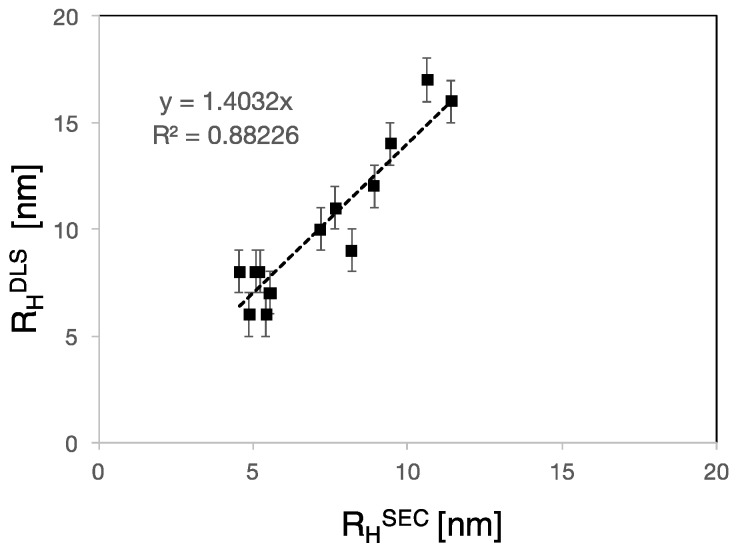
Hydrodynamic radii of PCEs as determined by DLS experiments as a function of those inferred by SEC measurements. The linear fit has slope of 1.4 and *R*^2^ of 0.88.

Therefore, using this relation, the hydrodynamic radius of PCEs based on the molar mass obtained using standard calibration, *R*_H_^SEC^, was calculated. Values obtained in this way are plotted in Figure 5 as a function of direct measurements of *R*_H_ performed by DLS for PCEs. 

The graph shows that both radii are proportional to each other. The fact that the proportionality constant is not equal to one can be understood by the differences between the nature of the determinations as well as the variation of the prefactor value in Equation (4) which depends on the polymer–solvent interactions.

Let us therefore consider a PCE molecule and a linear PEO/PEG of equal molar mass in solution. In principle, these two polymers should not elute at the same time, because the PCE has a much denser structure compared to the linear polymer and occupies a smaller volume in solution. In other words, for the same elution time (and *R*_H_) the mass of a linear polymer should be much smaller than that of a PCE. This highlights the limitations of inferring molar mass values of PCEs using linear standards for calibration of the SEC separation. In fact, the limited applicability of standard calibration for complex polymers has been debated for years, however this method is still largely used in industry to determine the molar mass of PCEs because of lack of suitable alternatives. 

The contraction of the size of the polymer with respect to a linear polymer of equal molar mass, due to the presence of the side chains, leads also to a reduced intrinsic viscosity. To highlight this, the Mark-Houwink (M-H) plot of each PCE [20], obtained by SEC-VISC, was examined. The M-H equation relates the intrinsic viscosity of a polymer to its molar mass: [η] = *KM^a^*. The M-H parameters, *K* and *a*, depend on the polymer type, solvent and temperature. The exponent *a* is related to the polymer topology and was found to be <0.4. This value is indicative of a branched structure, such as a comb-shaped polymer [20]. An example of a M-H plot and the values of the parameter *a* can be found in the Supplementary Materials (Figure S2, Table S2).

For what concerns the molar mass determination, Figure 4 shows that the molar masses inferred by SEC-MALS are more accurate than those determined by standard calibration. The discrepancies between the theoretical molar mass and the values determined by SEC-MALS can come from inaccuracies in the on-line determination of the refractive index increment (*d*n/*d*c), which changes along the elution volume. It may also be affected by the presence of small amounts of cross-linked fractions which may bias the MALS measurement.

### 4.2. PCE Conformation in Solution

Understanding the conformation of PCEs in solution is an important pre-requisite if molar masses are to be determined from SEC measurements. Therefore, we have examined whether Equation (1) accurately predicts the size in solution of PCEs. While previous reports do suggest this, in most cases the *n* values were back-calculated from the molar mass of the final PCE. This poses a problem since the molar mass of PCEs cannot be determined accurately.

In this paper we have used polymers prepared by grafting and for which the backbone size was previously determined. Therefore, together with an independent measurement of the grafting density it was possible to calculate the *n* independently of SEC measurements of the PCE itself. Additionally, it was possible to calculate both the expected *M*_w_ and *M*_z_ from the corresponding values of the backbone. In turn, this provided us with estimates of the weight and z-averages of the radius of gyration, *R*_G,w_ and *R*_G,z_, respectively.

**Figure 6:**
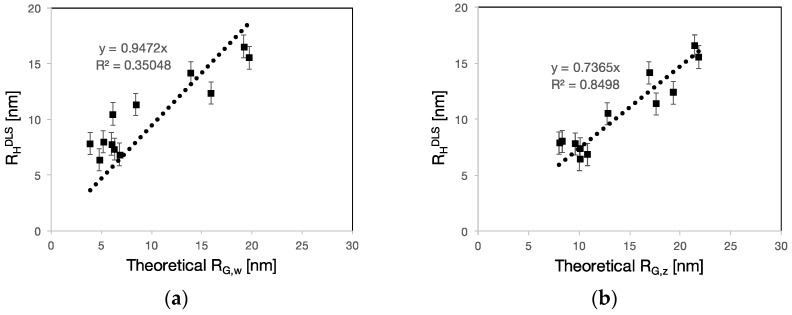
Experimental *R*_H_ as determined by DLS as a function of the: theoretical weight-average (**a**); and *z*-average, *R*_G_ (**b**). The intercept of the fits to both data sets was set equal to zero. The *z*-average radii of gyration have a better correlation coefficient (*R*^2^ = 0.85) with the experimental radii than the weight-average radii (*R*^2^ = 0.35).

When comparing these values with the *R*_H_ determined by DLS (Figure 6), we find that there is almost no correlation with *R*_G,w_ (*R*^2^ = 0.35). On the other hand, we find a very good correlation for *R*_G,z_. Note that both fits are forced to have an intercept equal to zero. 

This is not surprising since it is understood that scattering measurements are biased towards the presence of large molecules and that they are generally considered to provide information on *z*-average size rather than weight-average. 

This result indicates that the scaling relation in Equation (1) well captures the impact of the structural parameters on the hydrodynamic radius. It also shows that the prefactor in Equation (1) is close but not exactly equal to the one found by DLS. However, it must be considered that *R*_H_ and *R*_G_, although of the same order of magnitude, can have different absolute values. Indeed, in the case of most polymers, the ratio between *R*_G_ and *R*_H_ differs from unity [20].

These results also underline that the effect of dispersity on conformation of PCEs cannot be neglected. This is of particular importance when dealing with commercial PCEs, which have much larger molar mass distributions (*Đ* > 2.5) than those used in research. For broad-distributed PCEs, the theoretical *R*_G,w_ and *R*_G,z_ would have substantially different values. 

**Figure 7:**
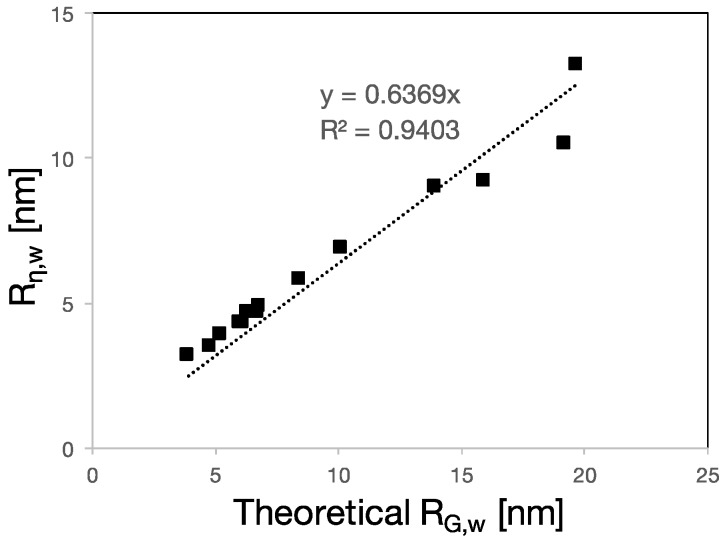
Weight-average viscometric radius calculated from the intrinsic viscosities measured by SEC-MALS as a function of theoretical weight-average radius of gyration. The linear fit has slope of 0.6 and *R*^2^ of 0.94. The error bars are included but not visible because they are smaller than the data markers.

Knowing that a PCE molecule possesses a reduced intrinsic viscosity compared to PEO/PEG of similar average molar mass due its compact structure, let us now consider the viscometric radius of the PCEs. This is understood to be related to a molar mass average which is rather close to the weight-average [20]. Consequently, the theoretical *R*_G_ with which it should be compared must be the one based on the *M*_w_ of the backbone, *R*_G,w_. The relation between the two radii is illustrated in Figure 7. 

**Figure 8:**
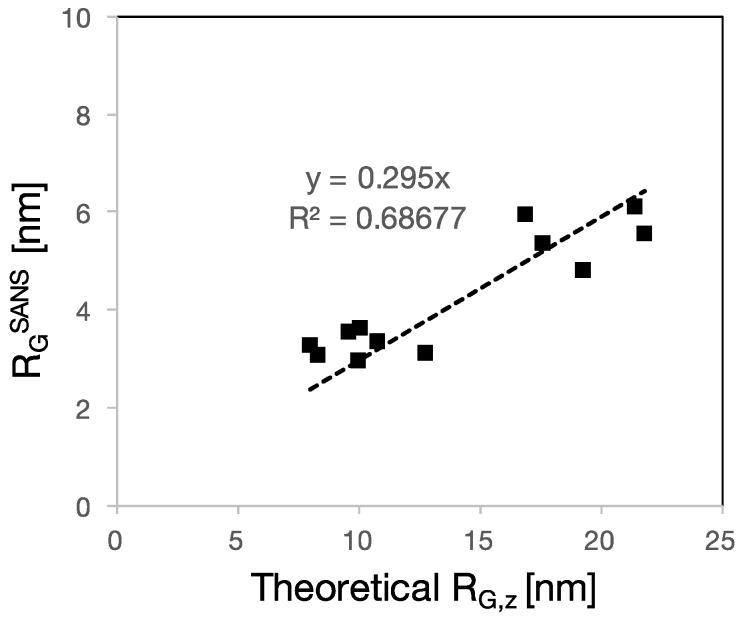
Radius of gyration of the PCEs determined by SANS as a function of the theoretical *R*_G,z_. The linear fit has slope of 0.3 and *R*^2^ of 0.7. The error bars are included but not visible because they are smaller than the data markers.

As shown in Figure 7, a good correlation between the viscometric radius and *R*_G,w_ was found. As for the correlation between the theoretical mass and the hydrodynamic radius (Figure 8), the slope is not unity. However, this also shows that Equation (1) well captures the molecular structure dependence of PCE conformation in solution.

Another characteristic dimension of the polymer in solution is the radius of gyration. We have determined this by SANS and the results are plotted versus the calculated *R*_G,z_ in Figure 8. 

As in the previous cases, we find a roughly linear correlation of which the slope is not unity. This indicates that the scaling behavior of Equation (1) is roughly followed by the experimental data. Although the correlation is not ideal, the proposed scaling law is able to qualitatively predict the radius of gyration of a number of polymer having different chemistries, synthetic pathways and molar masses. However, the magnitude of the experimental radius of gyration *R*_G_ is about three times smaller than the theoretical one. As mentioned, it is intrinsic to the derivation of scaling laws that they should well capture the exponents, but that their prefactors are not accurate. One reason for this is that single monomers and then blobs are taken to represent the persistence lengths in successive steps of the derivation of Equation (1) [28]. Any inaccuracy in these assumptions would not affect the exponents of the power laws, but would modify the value of the prefactor. What this means is that Equation (1) captures well the relative changes of size resulting from variation of the molecular structure parameters. However, it does not provide accurate quantitative values of those sizes.

Finally, we have calculated the ratio *R*_G_/*R*_H_, which gives an indication of the polymer architecture. For example, the ratio *R*_G_/*R*_H_ of a rod-like molecule is 2.36, while a polymer chain with a dense globular conformation exhibits a ratio of 0.77. Interestingly, our PCEs have *R*_G_/*R*_H_ values lower than 0.77 (Table 2). Such low *R*_G_/*R*_H_ values can be explained by *R*_H_ being more sensitive to the whole polymer chain, while *R*_G_ is more sensitive to the core of the structure. Such situations, leading to a *R*_G_/*R*_H_ ratio even lower than 0.77, are reported for example for core-shell structures [36,37]. 

### 4.3. Impact of Grafting Density on PCE Dispersity

Thus far, the contribution of the grafting density to the dispersity has been neglected and the *N* value was considered to be constant over the whole distribution of molar masses. However, this is not necessarily true.

By SEC analysis, it has been seen that the dispersity of the PCE is not the same as that of the backbone and in most cases is lower (Table 1). This should not be due to changes in the backbone during esterification, since any changes of it during that step would rather increase than decrease dispersity. It is neither expected to come from the MPEG since its dispersity is low. Most probably the decrease of dispersity during grafting implies that the side chains are not grafted equally onto backbones of different sizes. In particular, the grafting density would decrease with increasing size of the backbones, something which is consistent with the experimental observation that the grafting reaction becomes more challenging when longer backbones are used.

**Figure 9:**
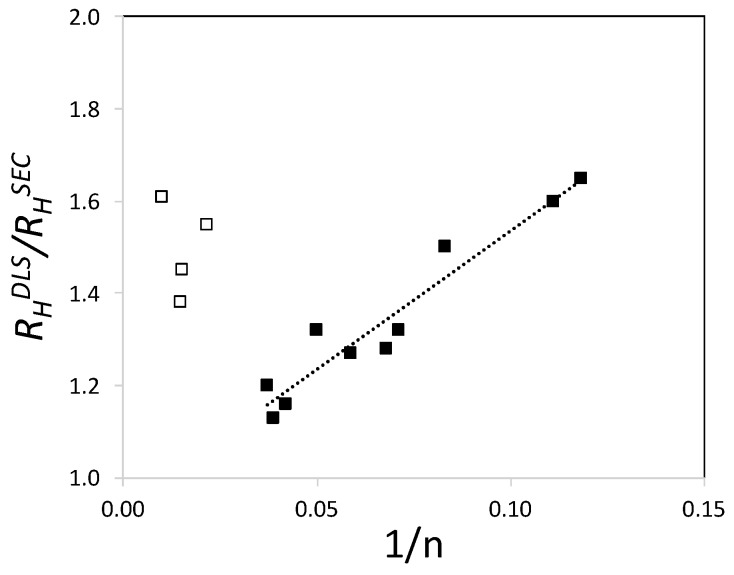
The *R*_H_^DLS^/*R*_H_^SEC^ ratio is plotted versus the inverse of *n*, the average number of grafted side chains. *R*_H_^DLS^ is the measured on PCEs by DLS and *R*_H_^SEC^ is the *z*-average value of the *R*_H_ distribution measured by SEC. The *R*_H_^DLS^/*R*_H_^SEC^ is only constant (as expected) for PCEs A1–A4 (empty squares), which were prepared with monodisperse backbones. For PCEs B1–B6 and C1–C4 (filled squares) prepared with polydisperse backbones, this is not the case.

This interpretation is further supported by the following consideration. Based on our previous results (Figure 5), the ratio *R*_H_^DLS^/*R*_H_^SEC^, where *R*_H_^DLS^ is the value measured on PCEs by DLS and *R*_H_^SEC^ is the *z*-average value of the *R*_H_ distribution determined by SEC, ought to be a constant independent of the polymer and its value should only reflect differences in the nature of the measurements. As shown in Figure 9, this expectation is only met for PCEs A1–A4, but not for the others. 

Importantly, PCEs A1–A4 are those of which the dispersity of the backbone and of the PCE are relatively narrow and similar to each other. This suggests that the variation in the ratio *R*_H_^DLS^/*R*_H_^SEC^ for polymers B1–B6 and C1–C4 is due to their greater dispersity.

In relation to this, we note that for these polymers, the ratio *R*_H_^DLS^/*R*_H_^SEC^ increases linearly with 1/*n* and that its highest value is similar to the average ratio of polymers A1–A4. In other words, PCEs produced with polydisperse backbones are most similar to ones produced with monodisperse backbones if the number of grafted side chains *n* is low (1/*n* is large). In turn, this indicates that there must be an inhomogenous grafting depending on the backbone size when polydisperse backbones are used. Further research is however needed, to back up this hypothesis.

### 4.4. Proposal for a Better Use of SEC in Characterizing PCEs

The results presented leave the open question of how to adequately determine the average molar mass of PCEs. 

The approach used in this work shows that it is possible to calculate the theoretical molar mass if PCEs are synthesized by esterification of a pre-formed backbones. This requires first determining the molar mass of the backbone using SEC and standard calibration with proper linear standards. The molar mass of the backbone, combined with the molar mass of the side chains and the grafting density, can then be used to calculate the theoretical molar mass. However, this approach does not give access to the molar mass distribution, but only to an average value of molar mass. At best, assuming that the grafting density is independent of the backbone size and that the side chains are rather monodisperse, the molar mass distribution would follow that of the backbone. However, we have shown that this is only true if the backbones are rather monodisperse.

Concerning direct characterization of PCEs with SEC, we found that SEC-MALS delivers relatively accurate molar masses. In literature, the standard practice consists of using the *d*n/*d*c value of PEO (0.135 mL/g) [38]. However, this approach has shortcomings since refractive index increments of PCEs also depend on the relative proportions of the different monomer types. Our results show that the online determination of the *d*n/*d*c value is essential to a good estimation of the molar mass. 

The two options mentioned above are useful to determine molar masses when new measurements are possible and compounds (backbone, side chains and PCEs) are still available. However, for reevaluating past measurements, typically done with standard calibration, other approaches must be considered. 

A tempting approach could be to convert the molar mass distribution obtained by SEC and PEO/PEG standard-based calibration into a distribution of hydrodynamic radii *R*_H_, using Equation (4) and shift it to overlap the distribution of theoretical radii of gyration as defined in Equation (1). However, as explained above, the overlap of the distributions would be legitimate only if the pre-formed backbones are relatively monodisperse. Otherwise, the grafting leads to a decreased dispersity attributed to a preferential grafting on the short rather than on the long backbones. Therefore, such a shifting cannot be done without making assumption on the grafting dispersity of PCEs, something which is beyond the scope of this paper.

### 4.5. Implications for the Structure–Property Characterization of PCEs

Finally, the implications of this work on the determination of the structure–property relations of PCEs are summarized in the scheme of Table 3. 

This scheme summarizes how to access the structural parameters *P*, *N* and *n* of PCEs. As mentioned in the introduction, *P* and *N* can be determined experimentally for PCEs synthesized both via esterification of a pre-formed backbone and copolymerization. The side chain length can be determined by proton NMR before or after the synthesis. It can also be determined by SEC before the synthesis. The side chain spacing can be obtained by proton NMR after synthesis, by analysis of the amount of non-grafted side chains after synthesis, or from the stoichiometry of the products used in copolymerization.

The parameter *n*, which is related to the length of the backbone, is the more delicate one, which most of this paper has addressed. Our results show that it can only be determined for PCEs synthesized by esterification, provided the pre-formed backbone is characterized before grafting by SEC. Then with the value of *N*, *n* can be determined. If this is not possible, MALS should be preferred over standard calibration when attempting to determine molar masses by SEC. However, one should bear in mind that values obtained are not fully reliable, as shown in Figure 4.

Fortunately, many structure–property relations of PCEs are dominated by *P* and *N*, but are relatively, if not completely, independent of *n*. This is, for example, the case for adsorbed layer thickness [28], sensitivity to sulfates [39], hydration retardation [19], and mass of polymer adsorbed per occupied area [39]. On the other hand, the parameter n does affect the adsorption equilibrium constant [16] and the conformation in solution (this paper).

## 5. Conclusions

In this work, the characterization of comb-shaped copolymers, namely PCEs, by SEC, DLS and SANS was presented. PCEs with different molecular structures and degree of dispersity were synthesized by esterification of a backbone of PAA or PMA of known length.

We highlighted the difficulty in determining the number of side chains per polymer (Table 3). More specifically, we showed that determination of molar mass of PCEs via the widely-used calibration with linear polymer standards is not adequate for such polymers. On the other hand, SEC-MALS allows the determination of more accurate molar mass values. Implications for the structure–property relations of these PCEs have also been highlighted (Table 3).

By combining SEC results with DLS and SANS, we were able to validate a theoretical model for the radius of gyration of PCEs based on molecular structural parameters. The model was shown to qualitatively capture the power law dependencies on molecular structure parameters of solution conformation. However, the numerical prefactor is not accurate, which means that, although the relative effect of changes in molecular structure can be well predicted, the absolute values are not. 

It is our hope that this paper has clearly highlighted the shortcomings of the widely used characterization of PCEs, namely SEC with standard calibration. We have shown how the independent determination of the molecular structure parameters can be used to calculate the theoretical molar mass and, eventually, to derive the molar mass distribution for rather monodisperse backbones. More importantly, this work also represents the first step to address the issue of the dispersity effect onto PCE properties in a broad sense.

## Figures and Tables

**Table 1:** Chemical characteristics and radii of the PCEs used in this work. The *Đ* value was calculated using the molar masses determined by standard calibration based on PAA, for the backbone (BB), and PEO/PEG, for the PCEs. The parameter *n*_w_ is calculated from the weight-average molar mass, *M*_w_, of the backbone. *R*_G,w_ and *R*_G,z_ are the radius of gyration calculated using *n*_w_ and *n*_z_, respectively. The parameter *n*_z_, is calculated from the *z*-average molar mass, *M*_z_, of the backbone.

PCE	BB type	*M*_w_^BB^ (g/mol)	*Đ*^BB^	*Đ*^PCE^	*n*_w_	*N*	*P*	*MM*^theor.^ (g/mol)	*R*_G,w_ (nm)	*R*_G,z_ (nm)
A1	PAA	23,000	1.8	1.6	64	5.0	45	150,700	15.9	19.3
A2	PAA	23,000	1.8	1.5	46	7.0	45	114,200	13.9	16.9
A3	PAA	26,000	1.3	1.5	96	3.7	45	218,000	19.2	21.4
A4	PAA	63,000	1.3	1.3	66	13.3	45	194,000	19.7	21.8
B1	PMA	5300	9.2	1.5	15	4.2	23	20,100	4.8	10.0
B2	PMA	9000	not available	1.5	26	4.1	23	34,500	6.7	-
B3	PMA	9000	not available	1.9	24	4.4	68	80,300	10.1	-
B4	PMA	5300	9.2	2.0	20	3.1	68	65,200	8.4	17.6
B5	PMA	5300	9.2	1.6	8	7.3	23	13,800	3.9	8.0
B6	PMA	5300	9.2	2.4	27	2.3	23	32,200	6.1	12.8
C1	PAA	4600	4.0	1.4	9	7.3	45	22,200	5.2	8.3
C2	PAA	4600	4.0	1.4	12	5.2	45	29,300	6.0	9.6
C3	PAA	4600	4.0	1.4	14	4.5	45	33,100	6.3	10.1
C4	PAA	4600	4.0	1.5	17	3.8	45	38,400	6.8	10.8

**Table 2:** Hydrodynamic, gyration and viscometric radii of PCEs, determined by DLS, SANS and SEC-VISC, respectively, and *R*_G_/*R*_H_ ratios. The errors are ±1 nm for *R*_H_^DLS^ and ±0.1 nm for *R*_G_^SANS^ and *R*_η_^SEC^.

PCE	*R*_H_^DLS^ (nm)	*R*_G_^SANS^ (nm)	*R*_η_^SEC^ (nm)	*R*_G_^SANS^/*R*_H_^DLS^
A1	12	4.8	9.2	0.40
A2	14	5.9	9.0	0.42
A3	17	6.1	10.5	0.36
A4	16	5.6	13.2	0.35
B1	6	3.0	3.5	0.49
B2	6	2.5	4.7	0.41
B3	9	4.7	6.9	0.52
B4	11	5.4	5.8	0.49
B5	8	3.3	3.2	0.41
B6	10	3.1	4.3	0.31
C1	8	3.1	3.9	0.39
C2	8	3.6	4.3	0.45
C3	7	3.6	4.7	0.52
C4	7	3.4	4.9	0.48

**Table 3:** Schematic representation of how to determine the PCE structural parameters *P*, *N* and *n* for PCEs synthesized by polymer analogous esterification and copolymerization.

PCE structure parameters	Characterization method	Esterification	Copolymeriazation
P	SEC, ^1^H-NMR	✔	✔
N	^1^H-NMR, HPLC, titration	✔	✔
n	SEC	✔ *	✗ **

* requires preliminary SEC measurements on backbone; ** not possible because the backbone is formed during synthesis.

## Supplementary Materials

The following are available online at www.mdpi.com/2073-4360/9/2/61/s1. Synthesis of PAA backbones by RAFT polymerization, Size-exclusion chromatography of PAA backbones, Proton-NMR of PCEs, Overlap concentration of PCEs, SEC-Viscometry, Dynamic light scattering, Small angle neutron scattering. Figure S1: ^1^H-NMR spectrum of a PCE (PCE C3), Figure S2: Mark-Houwink plot of a PCE (PCE A1), Figure S3: Hydrodynamic radius of PCEs measured by DLS at two different concentration, Figure S4: Examples of DLS curves of PCEs, Figure S5: Examples of SANS curves of PCEs, Table S1: Overlap concentration of PCEs, Table S2: M-H parameter *a* values for PCEs.
