# Supplementary material for: Characterization of Comb-Shaped Copolymers by Multidetection SEC, DLS and SANS
Source: Polymers (Basel). 2017 Feb 14;9(2):61. doi: 10.3390/polym9020061 (PMC6432231; doi:10.3390/polym9020061)
Supplement: Supplementary file 1 [file polymers-09-00061-s001.pdf]

Supplementary Materials: Characterization of Comb-Shaped Copolymers by Multidetection SEC, DLS and SANS

Giulia Gelardi, Nicolas Sanson, Gergely Nagy and Robert J. Flatt

1. Synthesis of PAA backbones by RAFT polymerization

1.1. Materials

Tert-Butyl acrylate (tBA, 98% Sigma-Aldrich, Buchs, Switzerland) was purified by vacuum distillation over CaH_2 before use. Azobisisobutyronitrile (AIBN, 98%, Sigma-Aldrich) was purified by recrystallization from ethanol. 2-Phenyl-2-propyl benzodithioate (99%, Sigma-Aldrich) was used without further purification as RAFT agent. Methanol (98%, Chemie Brunschwig AG, Basel, Switzerland), hydrochloric acid 37% (98%, Sigma-Aldrich), 1,4-Dioxane (≥99 %, Chemie Brunschwig AG), Chloroform-d (deuterium content 99.96%, Sigma-Aldrich) and deuterium oxide (deuterium content 99.9%, Sigma-Aldrich) were used as received.

1.2. Procedure

The monomer (tBA), the radical initiator (AIBN) and the RAFT agent were placed in a Schlenk tube and dissolved in 1,4-dioxane. The solution was degassed by 3 cycles of freeze-pump-thaw. The reaction was started by placing the flask into an oil bath at 65 °C and stopped after 24 h by immersion in liquid nitrogen. Different proportions of the reagent were used to obtain PAA with different lengths.

Precipitation of poly(tBA) in a solution of methanol and water (1:1 *v*/*v*) was carried out three times followed by drying under high vacuum. Poly(tBA) was hydrolyzed with HCl 37% in 1,4-dioxane, at 85 °C under reflux for six hours. The complete hydrolysis was confirmed by ^{1}H-NMR, in D_2O. After evaporation of the solvent and dissolution in water, the PAA was freeze-dried for one day.

2. Size-Exclusion Chromatography of PAA Backbones

A first estimation of the molar mass of the PAA backbones was done by determining the monomer conversion, using ^{1}H-NMR in $CDCl_3$, and assuming that the radical polymerization is controlled by the RAFT agent.

The molar mass distribution of PAAs was determined by SEC. A series of three PSS Suprema columns (0.8 × 30 cm, particle size 10 μm) with a pore size of 30 Å and 1000 Å was used. Data analysis was performed using WinGPC Software by PSS. The eluent used was $NaNO_3$ 0.2 M and the flow-rate used was 1 mL/min. Molar mass standards of polyacrylic acid (sodium salt) (1.25 kg/mol < M_P < 1100 kg/mol, Agilent Technologies, Santa Clara, CA, USA) were used to calibrate the SEC system. The absolute molar mass of the polyacrylic acids was also determined by SEC-MALS. The refractive index increment (dn/dc) was determined online. The molar mass values determined by the two methods were found to be in good agreement. This reflects the fact that standards and the analytes are chemically identical.

The accordance between the molar mass values determined by SEC and the theoretical ones together with the low polydispersity of the PAA (around 1.3) indicate that the polymerization was well controlled. Indeed, the RAFT-synthesized PAA show a polydispersity that is lower than that of the SEC standards.

3. Proton Nuclear Magnetic Resonance (^{1}H-NMR) of PCEs

The typical spectrum of a PCE is shown in Figure S1, together with the peak assignments.

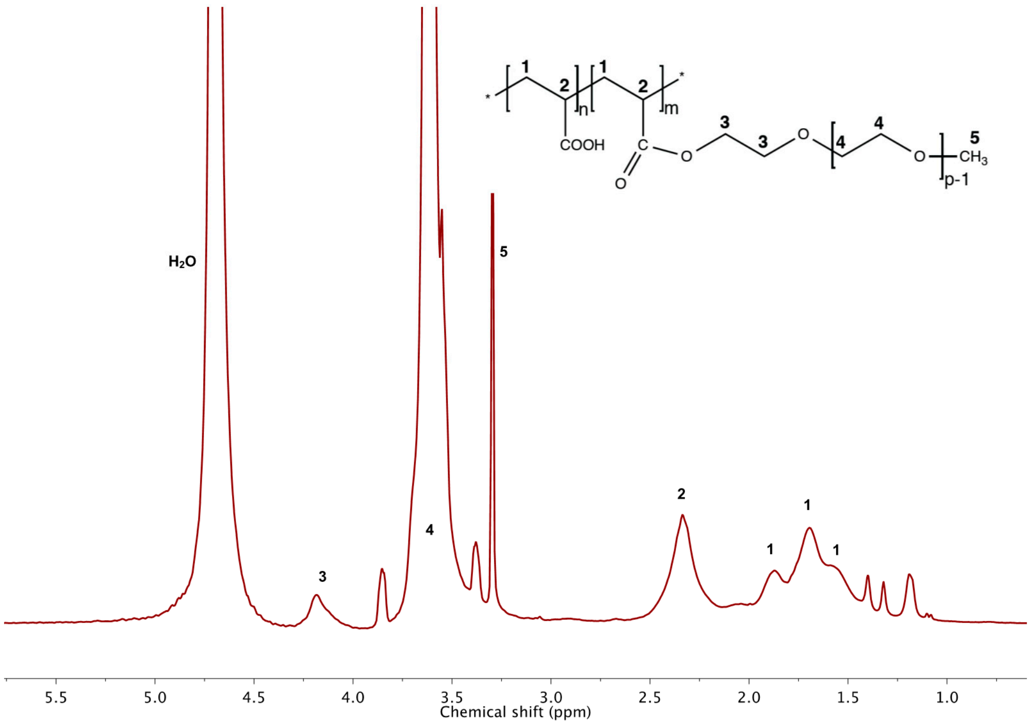

Figure S1. ^{1}H-NMR spectrum of a PCE (PCE C3).

^{1}H-NMR was used to confirm that esterification of the backbone has taken place. The protons of the first repeating unit of the MPEG have a different chemical shift with respect to those of the other repeating units. When an ester bond between the carboxylic function and the side chain occurs, a new peak, due to the protons next to the ester bond, appears in the NMR spectrum. Therefore, the formation of the ester bond was confirmed by the presence of the peak at about 4.2 ppm (peak 3), corresponding to the protons of the first repeating unit of the MPEG.

The grafting degree can be calculated from the ratio between the protons of the backbone (peaks 1 and 2) and those of the terminal methyl group of the side chain (peak 5).

4. Overlap Concentration of PCEs

A first estimate of the overlap concentration of the PCEs used in this work was calculated as the inverse of the intrinsic viscosity, measured by SEC-Viscometry and is noted C_η*. The "true" overlap concentration was calculated using the experimental values of R_G as determined by SANS and is noted C_{Rg}*. The overlap concentrations are reported in Table S1, together with the ratios between the overlap concentration C_{Rg}* and the concentrations used for DLS and SANS experiments, noted as C_{DLS} and C_{SANS}, respectively. The magnitude of these ratios confirm that all scattering experiments were performed in a dilute regime.

Table S1. Overlap concentrations of PCEs calculated from the intrinsic viscosity, C_η*, and from the R_G determined by SANS, C_{Rg}*. In the last two columns the ratio of C_{Rg}* to the concentrations used in the DLS and SANS experiments (C_{DLS} and C_{SANS}) are given, showing that the experiments were peformed in the dilute regime.

PCE	C_η* (g/L)	C_{Rg}* (g/L)	C_{Rg}*/C_{DLS}	C_{Rg}*/C_{SANS}
A1	29	540	270	54
A2	25	258	129	26
A3	28	520	260	52
A4	13	741	371	74

B1	69	295	148	30
B2	53	876	438	88
B3	39	271	135	27
B4	55	155	78	16
B5	64	152	76	15
B6	59	298	149	30
C1	53	160	80	16
C2	57	229	115	23
C3	47	239	120	24
C4	47	465	232	47

It has to be noted that both overlap concentrations are defined as mass of a polymer divided by its volume. However, since the polymer volume is defined differently in the two cases, the two overlap concentrations, C_η* and C_{Rg}*, have different magnitude. This difference depends on the polymer architecture, excluded volume effects and hydrodynamic interactions.

We estimated this factor by calculating C_η* using a analogous definition to that of C_{Rg}*. Using the viscometric radius R_η, instead of R_G, we obtained the following equation:

$$C_\eta^* = \frac{3M}{4\pi R_\eta^3}$$

From the definition of the viscometric radius in Equation (3) of the manuscript, we obtain:

$$C_\eta^* = \frac{5}{2}\frac{1}{[\eta]}$$

The new definition of C_η* leads to an average C_{Rg}*/C_η* ratio of 3.3 (with the exception of PCE A4, which can be considered an outlier). This ratio corresponds to a R_η/R_G ratio of 1.5, which is the average ratio of the measured radii of our polymers.

5. SEC-Viscometry

The Mark-Houwink plot is the plot of the intrinsic viscosity as a function of the molar mass of a polymer in a log-log scale. The M-H plot of a PCE is shown in Figure S2.

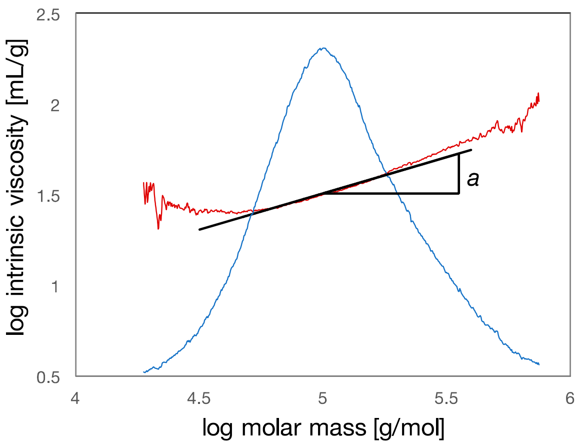

Figure S2. Mark-Houwink plot of a PCE (PCE A1). The blue line is the molar mass distribution of the polymer as determined by SEC-MALS (the axis of the detector signal is not shown). The red line is the intrinsic viscosity as a function of the molar mass. The black line is the fit to the intrinsic viscosity, whose slope is the M-H parameter *a*.

The values of the M-H parameter *a* determined from the Mark-Houwink plots are reported in Table S2.

Table S2. M-H parameter *a* values for PCEs.

PCE	M-H parameter *a*
A1	0.40
A2	0.36
A3	0.33
A4	0.40
B1	0.36
B2	0.35
B3	0.20
B4	0.17
B5	0.34
B6	0.30
C1	0.16
C2	0.10
C3	0.08
C4	0.03

6. Dynamic Light Scattering

The plot in Figure S3 reports the hydrodynamic radii as determined by DLS at two different concentrations, about 0.5 and 2 g/L.

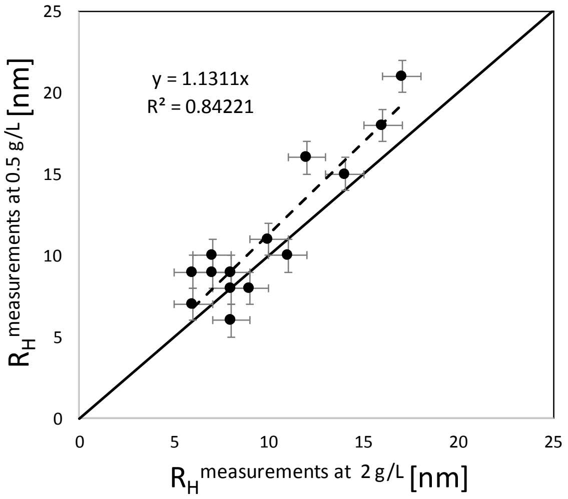

Figure S3. Hydrodynamic radii of PCEs as determined by DLS experiments on polymer solutions having a concentration of about 0.5 g/L and 2 g/L. The continuous line shows the 1:1 relation, while the discontinuous line is the best linear fit passing through the origin. Its slope is 1.13.

The results of Figure S3 show that the measured R_H does not depend on the concentration used meaning that the DLS measurements were carried out in the dilute regime.

Three examples of correlation functions are shown in Figure S4.

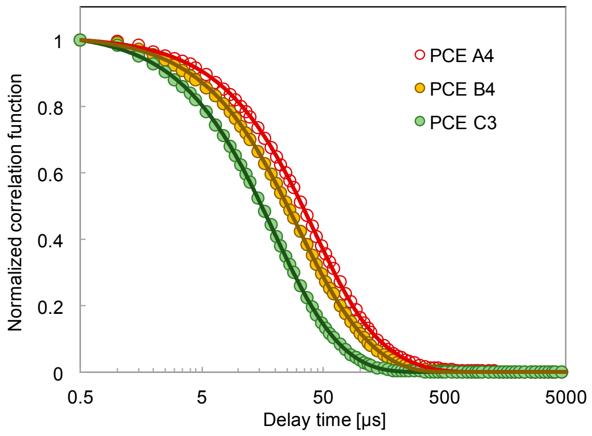

Figure S4. Correlation functions of the three types of PCEs used in this work (PCE A4, B4, C3) and corresponding fits.

7. Small angle Neutron Scattering

In Figure S5, the scattering intensity of three types of PCEs used in this work (PCE A2, B1, C3) is plotted as a function of the scattering vector *q*. The Debye fits are also shown.

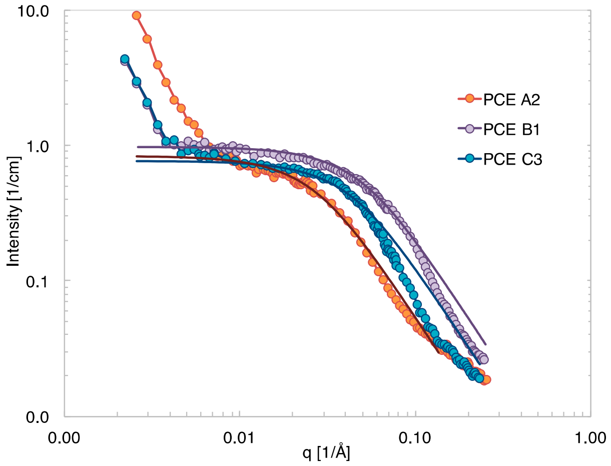

Figure S5. SANS curves of the three types of PCEs used in this work and corresponding fits.

At low *q* values, an increase of intensity can be observed. This is probably due to the presence of aggregates in the polymer solutions. In the mid-range of *q*, the typical profile of polymer chains can be observed.
